# Somatosensory mismatch response in young and elderly adults

**DOI:** 10.3389/fnagi.2014.00293

**Published:** 2014-10-27

**Authors:** Juho M. Strömmer, Ina M. Tarkka, Piia Astikainen

**Affiliations:** ^1^Department of Psychology, University of JyväskyläJyväskylä, Finland; ^2^Department of Health Sciences, University of JyväskyläJyväskylä, Finland

**Keywords:** aging, event-related potential, mismatch negativity, oddball condition, somatosensory

## Abstract

Aging is associated with cognitive decline and alterations in early perceptual processes. Studies in the auditory and visual sensory modalities have shown that the mismatch negativity [or the mismatch response (MMR)], an event-related potential (ERP) elicited by a deviant stimulus in a background of homogenous events, diminishes with aging and cognitive decline. However, the effects of aging on the somatosensory MMR (sMMR) are not known. In the current study, we recorded ERPs to electrical pulses to different fingers of the left hand in a passive oddball experiment in young (22–36 years) and elderly (66–95 years) adults engaged in a visual task. The MMR was found to deviants as compared to standards at two latency ranges: 180–220 ms and 250–290 ms post-stimulus onset. At 180–220 ms, within the young, the MMR was found at medial electrode sites, whereas aged did not show any amplitude difference between the stimulus types at the same latency range. At 250–290 ms, the MMR was evident with attenuated amplitude and narrowed scalp distribution among aged (Fz) compared to young (fronto-centrally and lateral parietal sites). Hence, the results reveal that the somatosensory change detection mechanism is altered in aging. The sMMR can be used as a reliable measure of age-related changes in sensory-cognitive functions.

## INTRODUCTION

It is suggested that the brain can rapidly and effortlessly learn the regularities in the stimulus environment and predict what should happen in the future (Wacongne et al., 2012). The brain is capable to detect sudden changes in the perceptual environment even without attentive resources. In aging, the change detection and predictive coding of the environmental events is gradually declined (Ruzzoli et al., 2012; see also Winkler and Czigler, 2012). There is a growing concern to understand widely the aspects of healthy aging as the world’s age breakdown is rapidly reversing; it is expected that in 2050 the world’s population at ages over 65 years will be 2.5 times that of the population at ages 0–4 years, the opposite ratio to 1950 (Haub, 2011).

On neurophysiological approach on aging, event-related potentials (ERPs) provide important indicators for pre-attentive sensory processing. The mismatch negativity (MMN) is a component of ERPs that occurs when the brain detects a change in a background of homogenous events (Näätänen, 1992). Further, its elicitation reflects predictive coding of the stimulus environment (Garrido et al., 2009; Wacongne et al., 2012). The MMN has been originally discovered in the auditory modality (Näätänen et al., 1978), but there is extensive evidence of the existence of its visual analog (for reviews see Kimura, 2012; Winkler and Czigler, 2012).

In normal aging, the MMN amplitude in the auditory (Cooper et al., 2006; Schiff et al., 2008; Kiang et al., 2009; Näätänen et al., 2012; Cheng et al., 2013) and visual (Tales et al., 2002; Lorenzo-Lopez et al., 2004) modalities have been shown to decrease gradually. In addition, the latency of the auditory MMN seems to prolong with age (e.g., Gaeta et al., 2001; Bertoli et al., 2002). These changes in MMN have been argued to indicate the shortening of the sensory memory duration and deficits in the encoding of the information due to age-related decline of the functional integrity of the central sensory processing (Pekkonen, 2000; Cooper et al., 2006). Importantly, the attenuation of MMN has been shown to reflect the deterioration in cognitive functions (Kisley et al., 2005; Foster et al., 2013). In these studies, decrease in the MMN to changes in intervals between the sounds correlated with poorer performance in cognitive tasks requiring executive function.

The reports of the somatosensory MMR (sMMR), a counterpart of auditory MMN, are sparse. Nonetheless, it is reliably obtained in adults (Kekoni et al., 1997; Shinozaki et al., 1998; Akatsuka et al., 2005; Spackman et al., 2010) and in healthy children (Restuccia et al., 2009). In these studies, the sMMR has been shown to be elicited in a response to changes or violations in stimulus site (different fingers) of an electric pulse, frequency or duration of a vibration burst or a within-pair inter-stimulus interval of stimulus pairs. In most studies, the sMMR has been elicited at about 100–200 ms after the stimulus onset over the fronto-central regions either as a negative or positive component, presumably depending on the direction of the generating dipole (Kekoni et al., 1997; Akatsuka et al., 2005). Spackman et al. (2007) found both a negative shift of a difference wave (deviant minus standard) at about 100–200 ms fronto-centrally contralateral to stimulus and a subsequent positive shift at about 150–250 ms with centro-parietal scalp distribution, despite of the stimulus site. Correspondingly, Restuccia et al. (2009) found in children a central negative shift of the difference wave at about 120–180 ms contralateral to stimulus, followed by a deflection at about 180–250 ms, albeit negative in polarity and distributed frontally contralateral to stimulus. In a tactile two-point discrimination task (Akatsuka et al., 2007a,b) the generators of the magnetic equivalent of the sMMR [the magnetic mismatch field (MMF)], peaking around 30–70 ms and 150–250 ms, were found in the primary and secondary somatosensory cortex contralateral to stimulus, respectively. Studies with intracranial recordings using vibrotactile stimulation have showed that the sMMR is localized on the postcentral gyrus on the cortex (Spackman et al., 2010; Butler et al., 2011).

Contrary to studies in the auditory and visual modalities, to our knowledge, there are no studies showing the effects of normal aging on the pre-attentive detection of somatosensory changes. Nevertheless, Bolton and Staines (2012) have studied age-related changes in somatosensory ERPs using tasks that require subject’s attentional resources. They suggested that in an attention-demanding somatosensory task age-related alterations in the attention mechanism are partly due to deficit in suppressing irrelevant sensory information. However, the elicitation of the sMMR does not rely on the subject’s attention or reactions and it is thus a potentially valuable tool for clinical purposes. Indeed, it has been showed that the sMMR can be used reliably for neurophysiological evaluation of tactile two-point discrimination (Akatsuka et al., 2007a) or the severity of a cerebellar dysfunction (Restuccia et al., 2007; Chen et al., 2014).

We recorded ERPs to electrical pulses with changes in the location of the stimuli in hand in two groups of subjects, young and elderly adults, while they were attending to a task in the visual modality. We hypothesize that the sMMR is elicited at the scalp regions representing the primary and secondary somatosensory cortices at about 100–250 ms after the stimulus onset as reported earlier (Shinozaki et al., 1998; Restuccia et al., 2009). We also hypothesize that the sMMR is attenuated in amplitude in aged compared to young similarly as in the auditory MMN.

## MATERIALS AND METHODS

### PARTICIPANTS

Electroencephalogram was collected from 22 young (22–36 years) and 14 elderly (66–95 years) Finns. All participants were right-handed volunteers with no self-reported neurological or psychiatric conditions. Five of the participants were discarded due to disrupted data (e.g., excessive movement during the recording). For the final data analysis there were 18 participants in the young adults group (22–29 years old, mean age 25 years, six female) and 13 participants in the elderly group (66–95 years old, mean age 75 years, nine female). The elderly group comprised of volunteers from the local organization of retired people recruited at their weekly meeting after an informative presentation of the study. The young adults group comprised university students recruited via e-mail. An informed written consent was obtained from each participant. The experiment was undertaken in accordance with the Declaration of Helsinki. The ethical committee of the University of Jyväskylä had approved the study.

### STIMULI AND PROCEDURE

During the recording, the subjects sat comfortably in a chair in a laboratory room. The subjects were instructed to ignore stimulation to the fingers and to be fully involved with a radio play, about which they were told to be asked questions afterward. The radio play was presented via loudspeaker placed about 50 cm above the subjects head with a volume subjectively comparable to normal speaking voice. The subjects were asked to fix their gaze at the cross on a computer screen placed about 1.5 m in front of the subject. The recording was video monitored from the room next to the subject’s room to control the subject’s sleepiness and movements during recording.

Electrical stimulation was generated with a constant current stimulator (Digitimer Ltd., model DS7A, Welwyn Garden City, UK). Electrical pulses of 200 μs in duration were delivered via conductive jelly moistened flexible metal ring electrodes (Technomed Europe Ltd., Maastrich, Netherlands) on the left forefinger and little finger (stimulating cathode above the proximal phalanx and anode above the distal phalanx). A piece of gauze was placed on the finger between electrodes to prevent conductivity between the two electrodes in the same finger. A run of 1000 stimuli was delivered with an inter-stimulus interval (ISI) of 500 ms. Frequently presented “standard” stimuli (probability 85%) were presented to one and rare “deviant” stimuli (probability 15%) to the other finger (forefinger and little finger). This assignment was counterbalanced between the subjects. Stimulus intensities were adjusted for each subject independently for both fingers to be twice the subjective sensory threshold, which was tested before recording. Overall, forefinger stimulus intensities were larger in the aged group (forefinger mean 5.5 mA, range 0.48–0.78 mA; little finger mean 4.4 mA, range 0.30–0.62 mA) than in the young group (forefinger mean 4.1 mA, range 0.28–0.56 mA; little finger mean 3.8 mA; range 0.24–0.48 mA) and larger to forefinger than to little finger within the both age groups: young *t*_17_ = 3.50, *p* = 0.003, *d* = 0.500; aged *t*_12_ = 4.10, *p* = 0.003, *d* = 0.208. One-way ANOVA showed a significant difference between the age groups in forefinger stimulus intensity (*F*_1,29_ = 21.24,* p* < 0.001), but no significant difference between little finger stimulus intensities (*F*_1,29_ = 3.60,* p* = 0.068).

### EEG ACQUISITION

Electroencephalogram was recorded with Brain Vision Recorder software (Brain Products GmbH, Munich, Germany) at 30 scalp locations. Ag/AgCl electrodes were placed on the electrode cap (Easy Cap QA40) according to the modified International 10–20 System at FP1, FP2, Fz, F3, F4, F7, F8, FC1, FC2, FC5, FC6, T7, T8, C3, C4, Cz, CP1, CP2, CP3, CP4, CP5, CP6, Pz, P3, P4, P7, P8, Oz, O1, and O2. Linked left and right mastoid electrodes served as a reference for all electrodes. The ground electrode was placed in the middle of the forehead. Eye movements and blinks were measured from bipolar electrodes placed one above the left eye and another lateral to the right orbit. The signal was amplified (Brain Vision QuickAmp), filtered with a band pass of 0.1–100 Hz and stored on hard disk at a sampling rate of 1000 Hz.

### DATA PROCESSING

The data were analyzed with Brain Vision Analyzer 2.0 software (Brain Products GmbH). The signals from the electrodes were first filtered with a band pass of 0.1–20 Hz (24 dB/octave roll off) and divided in stimulus onset-locked segments from -100 to +500 ms by stimulus type (deviant stimulus and standard stimulus immediately preceding the deviant stimuli). Segments with signal amplitude exceeding ±90 μV from the averaging and any recording channel were omitted from the further analysis. The pre-stimulus baseline was corrected by the mean amplitude between -100 to 0 ms. An average of 122 of standard (min = 79, max = 149, median = 131) and an average of 123 deviant (min = 64, max = 148, median = 128) trials were available for the further analysis from each individual.

Visual inspection indicated amplitude differences between standard and deviant responses for P50 and N80 components. Accordingly, the maximum peak amplitude value at C4 electrode (Shinozaki et al., 1998) and its latency were calculated within a time window of 30–80 ms and 40–110 ms after the onset of the stimulus for P50 and N80, respectively. To compare difference between the stimulus types and between the age groups, statistical analysis of ERP peak amplitudes of P50 and N80 were performed in repeated measures multivariate analysis of variance (MANOVA) with factors of Stimulus type (standard, deviant) and Age group (young, aged). In addition, visual inspection revealed MMR-like differential responses at 180–220 ms and 250–290 ms after the stimulus onset, labeled as early and late MMR, respectively. Accordingly, mean amplitude values from these time windows at nine electrode sites (FC1, Fz, FC2, C3, Cz, C4, P7, Pz, P8) were calculated. The selection of the electrode sites were based on the visual inspection of grand averaged scalp topography maps and previous findings on sMMR (Restuccia et al., 2009). MANOVA with within-subjects factors of Stimulus type (standard, deviant), Laterality (left: FC1, CP1, P7; medial: Fz, Cz, Pz; right: FC2, CP2, P8), Anteriority (frontal: FC1, Fz, FC2; central: CP1, Cz, CP2; parietal: P7, Pz, P8) and between-subjects factor of Age group (young, aged) were applied. Whenever group differences were found, differential ERPs (deviant minus standard responses) were calculated separately for both age groups and analysis of variances (ANOVA) was performed to compare differential responses between the groups. Finally, Pearson’s correlation coefficients, controlled with age, were computed separately within the each age group to examine the relationship between the stimulus intensity and differential ERPs.

Effect size estimates are described as partial eta squared ($\eta_{p}^{2}$) scores for MANOVA and Cohen’s *d* for *t*-tests. Paired samples *t*-tests were two tailed. The threshold for statistical significance was *p* < 0.05. Since focusing on the processing of different stimulus types, here we report only the main effects and interaction effects of MANOVA including the factor of Stimulus type.

## RESULTS

**FIGURE 1:**
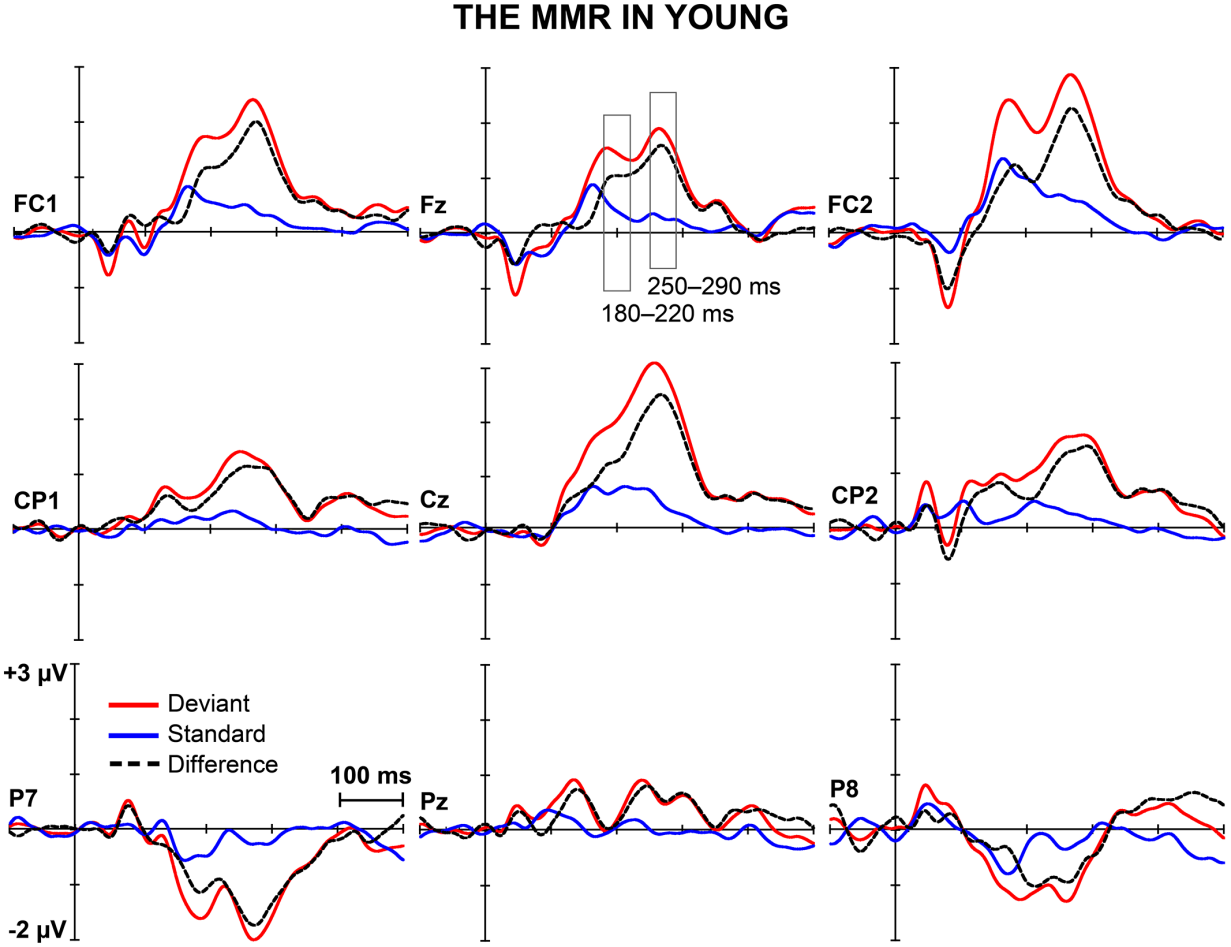
**The MMR in young.** The grand-averaged waveforms to deviant and standard stimuli and a differential waveform (deviants minus standards) within young at electrode sites analyzed for the early and the late MMR.

**FIGURE 2:**
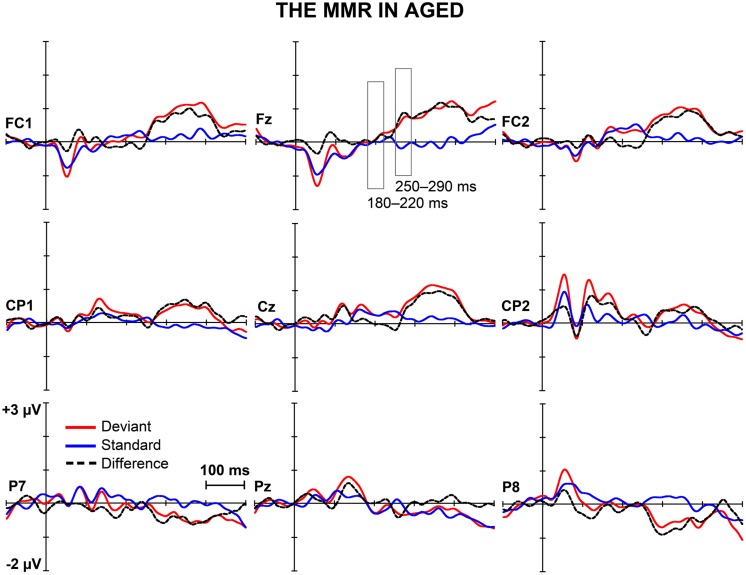
**The MMR in aged.** The grand-averaged waveforms to deviant and standard stimuli and a differential waveform (deviants minus standards) within aged at electrode sites analyzed for the early and the late MMR.

**FIGURE 3:**
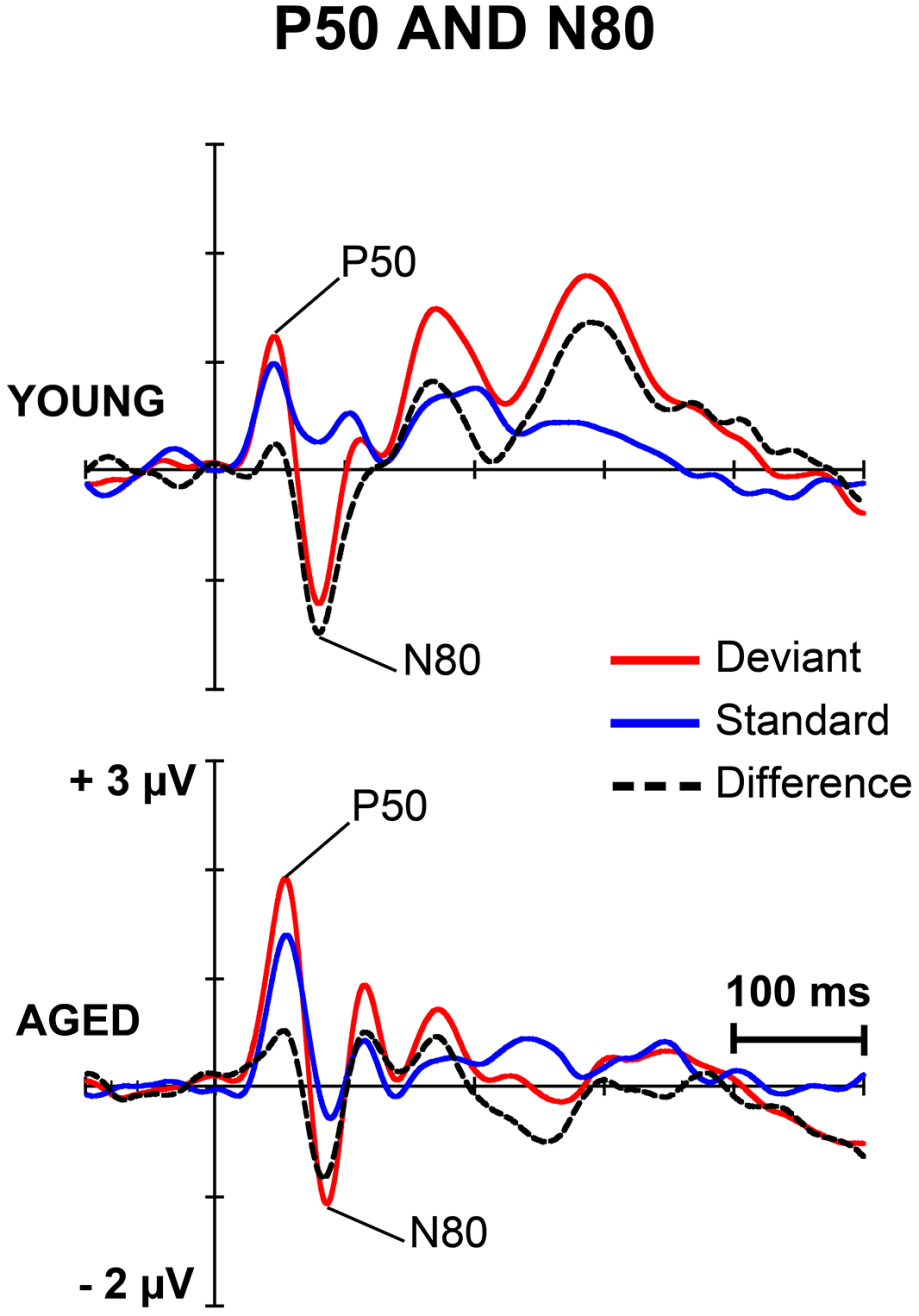
**The grand-averaged waveforms to deviant and standard stimuli and a differential waveform within each age group at electrode C4, where P50 and N80 were analyzed**.

**Figures 1** and **2** depict the grand-averaged waveforms to deviant and standard stimuli and a differential waveform within each age group on analyzed electrode sites. The grand-averaged waveforms for P50 and N80 on C4 are shown in **Figure 3**.

### P50

For P50 amplitude, a MANOVA showed a significant main effect of Stimulus type (*F*_1,29_ = 6.13,* p* = 0.019, $\eta_{p}^{2}$ = 0.175), but neither an interaction effect between Stimulus type and Age group (*F*_1,29_ = 1.35, *p* = 0.254, $\eta_{p}^{2}$ = 0.045) nor any other interaction effect with Stimulus type. Mean difference between the responses to deviant and standard stimuli was 0.42 μV (95% confidence interval 0.048–0.787 μV).

For P50 latency, all the effects were non-significant including the main effect of Stimulus type (*F*_1,29_ = 0.12, *p* = 0.730, $\eta_{p}^{2}$ = 0.004) and the interaction effect of Stimulus type × Age group (*F*_1,29_ = 0.20, *p* = 0.656, $\eta_{p}^{2}$ = 0.007).

### N80

An effect of Stimulus type was significant (*F*_1,29_ = 15.17,* p* = 0.001, $\eta_{p}^{2}$ = 0.343). Mean difference between the responses to deviant and standard stimuli was -1.13 μV, 95% confidence interval -1.695 to -0.570 μV). An interaction effect of Stimulus type × Age group was non-significant (F_1,29_ = 1.02, *p* = 0.322, $\eta_{p}^{2}$ = 0.034) as were the other interaction effects. Negative correlations between the N80 amplitude to deviant stimuli and the stimulus intensity to forefinger (*r* = -0.602, *p* < 0.001) and to little finger (*r* = -0.386, *p* = 0.035) were found.

For the latency, no significant main effect of Stimulus type (*F*_1,29_ = 1.23,* p* = 0.277, $\eta_{p}^{2}$ = 0.041) was found, but there was a significant interaction effect between Stimulus type and Age group (*F*_1,29_ = 6.73,* p* = 0.015, $\eta_{p}^{2}$ = 0.188). Thus, the standard and deviant stimulus responses were compared separately within each age group. Among young, the response latency to deviant stimuli was prolonged compared to that to standard stimuli (mean latencies 80.8 and 75.2 ms, respectively), *t*_17_ = 2.45, *p* = 0.025, *d* = 0.486 (mean difference 5.6 ms, 95% confidence interval 0.8–10.3 ms). Within aged no difference between the peak latencies to different stimulus types was found (*t*_12_ = 1.39, *p* = 0.189, *d* = 0.402, mean difference -2.2 ms, 95% confidence interval -5.7 to 1.3 ms; mean latency for the deviant responses were 85.9 and 88.2 ms for the standard responses). Further, an ANOVA showed that responses to standards (*F*_1,29_ = 10.02,* p* = 0.004), but not to deviants (*F*_1,29_ = 3.15,* p* = 0.088), were prolonged in aged compared to young.

### THE MMR

**FIGURE 4:**
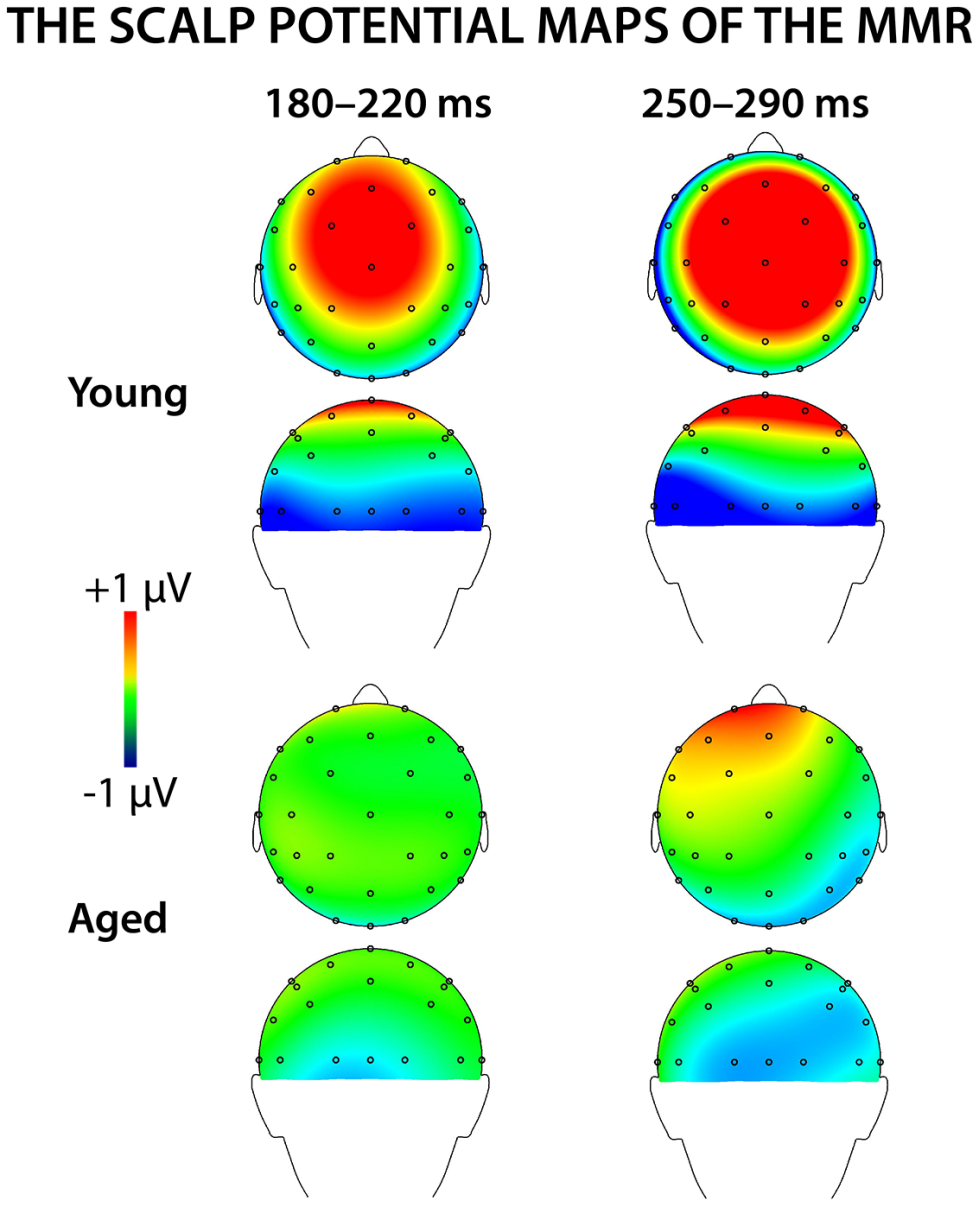
**The voltage distribution maps for the early and late MMR in young and aged**.

**Figure 4** shows mean scalp potential maps for the differential responses (deviant minus standard stimulus responses) at the analyzed latency ranges. Correlation analysis (Pearson’s, controlled with age, Bonferroni-adjusted) showed no correlation between the stimulus intensities to fingers and the amplitude values of differential responses for early or late MMR.

#### Early MMR: 180–220 ms

A MANOVA showed a significant main effect of Stimulus type (*F*_1,29_ = 5.75,* p* = 0.023, $\eta_{p}^{2}$ = 0.165) and interaction effects of Stimulus type × Laterality (*F*_2,28_ = 7.92,* p* = 0.002, $\eta_{p}^{2}$ = 0.361) and Stimulus type × Centrality (*F*_1,28_ = 5.13,* p* = 0.013, $\eta_{p}^{2}$ = 0.268), indicating inequality in scalp distributions of the ERP amplitudes to the different stimulus types (i.e., a mismatch response).

In addition, significant interaction effects of Stimulus type × Age group (*F*_1,29_ = 5.27,* p* = 0.029, $\eta_{p}^{2}$ = 0.154) and Stimulus type × Laterality × Age group (*F*_2,28_ = 4.08, *p* = 0.028, $\eta_{p}^{2}$ = 0.226) were found, revealing unequal responses between the age groups and non-homogenous scalp distribution of the MMR between young and aged. Since Stimulus type × Centrality × Laterality × Age group (*F*_4,26_ = 2.13,* p* = 0.106, $\eta_{p}^{2}$ = 0.247) and Stimulus type × Centrality × Age group (*F*_2,28_ = 3.01,* p* = 0.066, $\eta_{p}^{2}$ = 0.177) showed no significant effect, amplitude values were averaged over left, medial and right electrode sites, and responses to standard and deviant stimuli were compared in paired samples *t*-tests separately within each age group. Within young, responses to deviant stimuli differed significantly to those of standard stimuli at medial electrode sites, *t*_17_ = 3.57, *p* = 0.002, *d* = 1.033. Differential responses to the two stimulus types at right hemisphere electrode sites, *t*_17_ = 2.09, *p* = 0.052, *d* = 0.563, and left hemisphere electrode sites, *t*_17_ = 1.99, *p* = 0.063, *d* = 0.477, did not reach the significance. Among aged the difference in amplitude between the ERPs to deviants and standards were not evident at any of the analyzed averaged electrode sites (medial: *t*_12_ = 0.51, *p* = 0.616, *d* = 0.180; left: *t*_12_ = 0.35, *p* = 0.736, *d* = 0.093; right: *t*_12_ = 0.03, *p* = 0.981, *d* = 0.010; **Table 1**).

**Table 1:** Latency range of 180–220 ms.

Electrode pool	*t*	*P* (2-tailed)	*d*	Mean difference (μV)	95% CI lower	95% CI upper
Left/young	1.99	0.063	0.477	0.23	-0.014	0.479
Left/aged	-0.35	0.736	0.093	-0.02	-0.168	0.122
Medial/young	3.57	0.002	1.033	0.77	0.315	1.224
Medial/aged	0.51	0.616	0.180	0.06	-0.181	0.292
Right/young	2.09	0.052	0.563	0.26	-0.002	0.532
Right/aged	-0.03	0.981	0.010	-0.001	-0.293	0.286

*Post hoc tests (paired samples *t*-tests) for the interaction effect Stimulus type × Laterality × Age group. CI, confidence interval. *d*, Cohen’s *d*.*

The MMR was different in amplitude between the age groups at medial electrode sites (*F*_1,29_ = 6.96,* p* = 0.013), but not at left (*F*_1,29_ = 2.93,* p* = 0.097), neither at right (*F*_1,29_ = 2.05,* p* = 0.163) electrode sites. The differential mean amplitudes were larger in young (left 0.23 μV, middle 0.77 μV, right 0.26 μV) compared to aged (left -0.02 μV, middle 0.06 μV, right -0.001 μV; **Table 1**).

#### Late MMR: 250–290 ms

**FIGURE 5:**
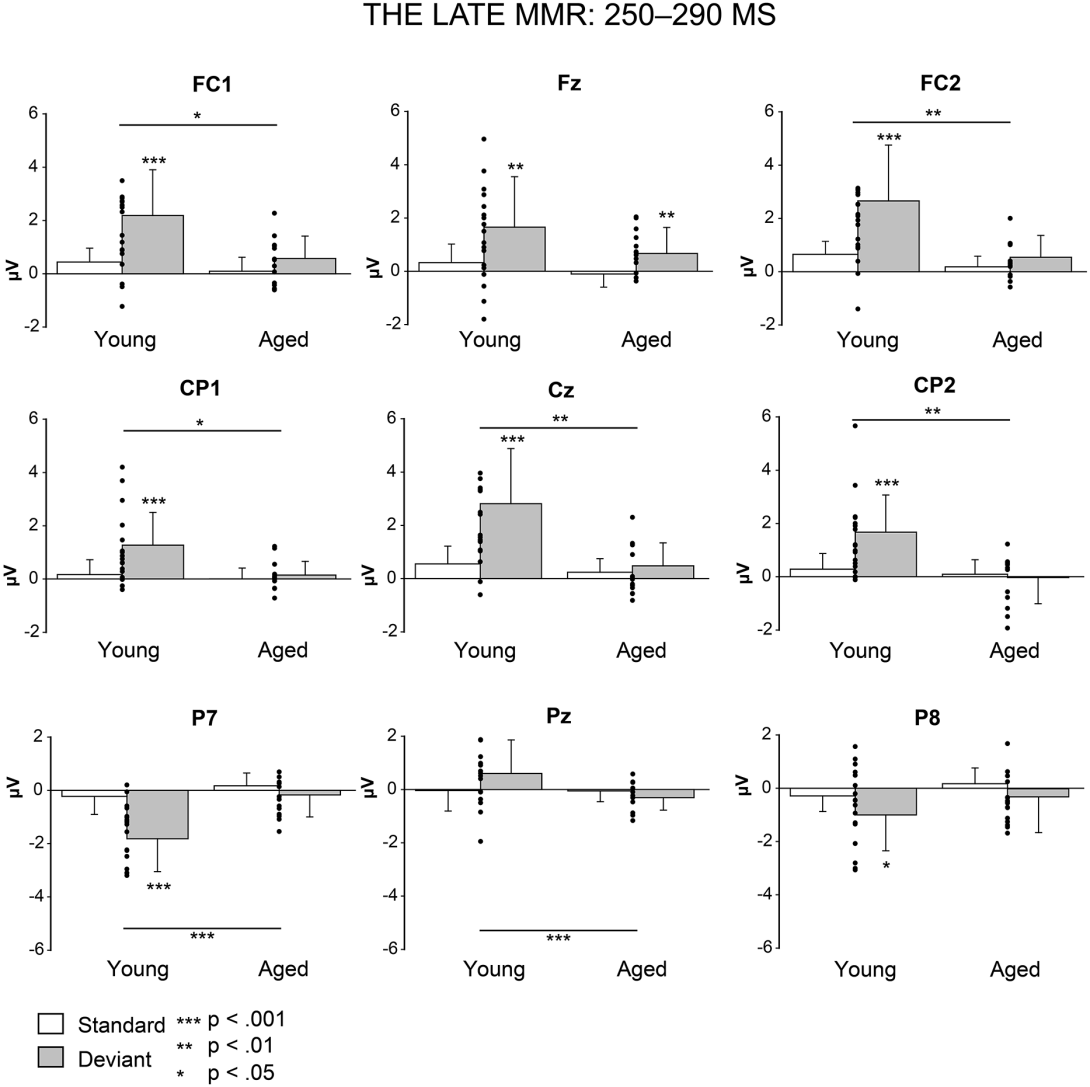
**Mean amplitudes and standard deviations to deviant and standard stimuli responses on analyzed electrode sites in young and aged.** Values of the differential response (deviants minus standards, i.e., MMR) of the individual participants’ are shown as scatterplots.

**Figure 5** shows the mean amplitudes to deviant and standard responses, standard deviations and individual participants’ amplitudes of the differential responses (deviant minus standard) in both age groups. A MANOVA revealed a significant main effect of Stimulus type (*F*_1,29_ = 13.31,* p* = 0.001, $\eta_{p}^{2}$ = 0.315) and significance for the all interaction effects including the factor of Stimulus type: Stimulus type × Laterality (*F*_2,22_ = 11.33,* p* = 0.0001, $\eta_{p}^{2}$ = 0.447), Stimulus type × Centrality (*F*_2,22_ = 9.22,* p* = 0.001, $\eta_{p}^{2}$ = 0.397), Stimulus type × Laterality × Centrality (*F*_4,26_ = 4.30,* p* = 0.008, $\eta_{p}^{2}$ = 0.398), Stimulus type × Age group (*F*_1,29_ = 8.96,* p* = 0.006, $\eta_{p}^{2}$ = 0.236), Stimulus type × Laterality × Age group (*F*_2,28_ = 5.90,* p* = 0.007, $\eta_{p}^{2}$ = 0.296), Stimulus type × Centrality × Age group (*F*_2,28_ = 5.61,* p* = 0.009, $\eta_{p}^{2}$ = 0.286), and Stimulus type × Laterality × Centrality × Age group (*F*_4,26_ = 4.41,* p* = 0.007, $\eta_{p}^{2}$ = 0.404). The 4-tailed interaction indicates an unequal scalp distribution of the MMR between the age groups. The subsequent paired samples *t*-tests comparing the standard and deviant stimulus responses were applied separately on each analyzed electrode site and for both age groups (**Table 2**). Within young the difference in amplitude between the ERPs to deviant and standard stimuli were significant at FC1, Fz, FC2, CP1, Cz, CP2, P7, P8 (*t*_17_ = 6.28–4.66, *p* < 0.001–0.045, *d* = 0.690–1.575). Within aged, instead, ERPs to deviant and standard stimuli differed significantly only at Fz (*t*_12_ = 3.53, *p* = 0.004, *d* = 0.972).

**Table 2:** Latency range of 250–290 ms.

Electrode site/age group	*t*	*P* (2-tailed)	*d*	Mean difference (μV)	95% CI lower	95% CI upper
FC1/young	4.30	<0.001	1.375	1.75	0.890	2.602
FC1/aged	2.00	0.069	0.685	0.48	-0.043	-0.017
Fz/young	3.24	0.005	0.932	1.33	0.464	2.199
Fz/aged	3.53	0.004	0.972	0.77	0.294	1.245
FC2/young	4.36	<0.001	1.320	2.01	1.035	2.979
FC2/aged	1.74	0.107	0.558	0.36	-0.090	0.807
CP1/young	3.56	0.002	1.154	1.10	0.450	1.756
CP1/aged	0.93	0.370	0.308	0.14	-0.191	0.477
Cz/young	4.66	<0.001	1.473	2.26	1.237	3.285
Cz/aged	0.93	0.370	0.337	0.24	-0.320	0.798
CP2/young	4.11	0.001	1.301	1.39	0.677	2.107
CP2/aged	-0.46	0.651	0.155	-0.12	-0.704	0.457
P7/young	-6.28	<0.001	1.595	-1.59	-2.122	-1.054
P7/aged	-1.80	0.097	0.511	-0.34	-0.761	0.072
Pz/young	2.05	0.057	0.612	0.64	-0.020	1.298
Pz/aged	-1.74	0.107	0.571	-0.25	-0.564	0.063
P8/young	2.00	0.045	0.690	-0.72	-1.414	-0.017
P8/aged	-1.70	0.114	0.507	-0.46	-1.058	0.130

*Paired samples *t*-tests (deviant vs. standard stimulus responses). CI, confidence interval. *d*, Cohen’s *d*.*

The MMR (deviant minus standard differential response) was different in amplitude between the age groups at FC1 (*F*_1,29_ = 5.91,* p* = 0.022), FC2 (*F*_1,29_ = 8.31,* p* = 0.007), CP1 (*F*_1,29_ = 6.12, *p* = 0.019), Cz (*F*_1,29_ = 10.87,* p* = 0.003), CP2 (*F*_1,29_ = 10.87,* p* = 0.003), P7 (*F*_1,29_ = 13.38,* p* = 0.001), Pz (*F*_1,29_ = 5.24,* p* = 0.030), but not at Fz (*F*_1,29_ = 1.17,* p* = 0.289) neither at P8 (*F*_1,29_ = 0.307,* p* = 0.584; **Table 2**).

## DISCUSSION

We recorded ERPs to changes in somatosensory stimuli, i.e., electrical pulses to different fingers in healthy young and elderly adults in a passive oddball condition. The sMMR was positive in polarity and elicited at two latency ranges in young: centro-parietally at 180–220 and fronto-centrally at 250–290 ms after the stimulus onset. In aged, the sMMR was attenuated and elicited only at the latter latency window with reduced scalp distribution compared to young. While in elderly the sMMR was evident only at Fz, it was found widely at fronto-central electrodes in young participants.

The sMMR to location changes has been found earlier by Shinozaki et al. (1998) in young adults. In addition, there are corresponding results in children (Restuccia et al., 2009). Shinozaki et al. (1998) found a central positive deflection to middle or index finger deviants at 100–200 ms post-stimulus, compatible to the early sMMR found in the present study. However, they did not report the following frontal positivity that was found in our study both in young and aged, probably owing to linked ear lobes reference used in their study compared with the average reference of the present study. Chen et al. (2014) argued in light of their findings that the sMMR is less sensitive to changes in location than to duration: they reported fronto-central negativity to vibrotactile duration deviants at 150–250 ms, but did not find sMMR to location changes. They suggested that the sMMR to spatially separated stimuli was absent due to relatively high age (mean 57.5 years) of their participants or too low stimulus intensity used in their study. In our data the late sMMR were found in aged despite of notably older age of the participants (mean age 75 years) than in the study of Chen et al. (2014), albeit the stimulus intensities were higher in our study, too. However, we found no correlation between the stimulus intensity and the sMMR amplitude though we did not specifically test extreme stimulus intensities.

In addition to the early sMMR (180–220 ms) found in young adults there was also a differential response at later latency range (250–290 ms) which was significant in both age groups. A few earlier studies have found sMMR in two latency ranges, albeit the findings seem somewhat discrepant. Akatsuka et al. (2005) reported a sMMR to temporal discrimination deviants eliciting an early negativity and a positive deflection at 100–200 ms post-stimulus. Spackman et al. (2007) found instead a negative fronto-central shift of a difference wave at 100–200 ms followed by a centro-parietal positive shift at 150–250 ms to vibrotactile presented changes in duration and frequency. The latter was suggested to reflect a process that is specific to sensory discrimination in the somatosensory modality. Similarly, Butler et al. (2011) reported a sMMR of negative polarity to duration changes approximately peaking at 145 ms followed with a fronto-central sMMR of positive polarity peaking at 235 ms post-stimulus. The late sMMR found in the present study seems to be similar in scalp topography and only slightly later in latency compared to the sMMR reported by Butler et al. (2011).

To our knowledge, the present study is the first to show the reduction of the sMMR in healthy aging. The results are in line with the findings of the auditory MMN. A recent meta-analysis concluded that the MMN to frequency and duration changes considerably declines in normal aging (Cheng et al., 2013). There is evidence from auditory studies linking the reduction of the MMN amplitude to decline in modality specific cognitive processing (Kisley et al., 2005; Mowszowski et al., 2012; Foster et al., 2013) and amnestic mild cognitive impairment (Lindin et al., 2013). Also the visual MMN to changes in motion direction and object form have been reported to diminish in aging, analogously to the auditory MMN (Tales et al., 2002; Lorenzo-Lopez et al., 2004). In addition, it has been shown that the latency of the auditory MMN to frequency (Gaeta et al., 2001) and temporal (Bertoli et al., 2002) changes is prolonged in aging.

A possible explanation for the aging-related diminution of the MMR may also lie behind disturbed predictive coding of sensory information. The predictive coding models presume that the brain continuously updates an internal model of environment by synaptic plasticity to predict the causes of sensory input; the MMN represents an inconsistency between the predicted sensory input (repetitive standards) and the unlearned (deviant) stimulus (Friston, 2005; Garrido et al., 2009; Wacongne et al., 2012). Further, *N*-methyl-D-aspartate (NMDA) receptor, a predominant controller of synaptic plasticity and memory function, have been proposed to have a fundamental role in predictive coding and the MMN generation (Tikhonravov et al., 2008, 2010; Wacongne et al., 2012). Aging-related deficiency in NMDA function may thus at least partly explain the MMN reduction in aging (Muller et al., 1994; Näätänen et al., 2011). It is also possible that in aging, predictive coding of stimulus characteristics is interfered by declined gating of sensory inputs (Chao and Knight, 1997) due to reduced inhibitory function (Reuter-Lorenz and Park, 2010; Bolton and Staines, 2012) in the sensory cortices (David-Jurgens and Dinse, 2010; Cheng and Lin, 2013). The assumption of age-related deficit in suppression of irrelevant sensory stimuli cannot be tested in the present data which was not designed to study the above-mentioned mechanism (see, e.g., Kisley et al., 2005). Nevertheless, we found age-related prolongation of the N80 latency (see **Figure 3**) indicating that aging might also have an effect on early sensory processing that precede the higher order sensory-cognitive functions.

There are limitations in the present study that future studies can address. First, the relationship between the age-related reduction of the sMMR and the cognitive function should be confirmed by using neuropsychological test batteries and carefully controlled demographic information (e.g., lifestyle factors and educational level). Second, we did not apply any control condition in order to investigate the underlying neural mechanism of sMMR (for a review of underlying mechanism of the auditory MMN, see Näätänen et al., 2005). Third, a low amount of sensors used in the EEG recording of the present study did not enable the application of source localization in the present data. Thus, no inferences of the processing hierarchy or pathways of the sMMR generation in the cortex can be made. Finally, although the preliminary results of the present study clearly demonstrate age-related effects to somatosensory deviance detection, our findings should be confirmed in future studies with larger sample sizes, and wide-ranging age range of participants, in order to determine whether the effects of aging on sensory-cognitive processing are constant within the adult life span.

In conclusion, the present study showed that the sMMR to location changes is sensitive to aging. The sMMR was attenuated in amplitude and prolonged in latency in aged compared to young adults. The findings provide new knowledge for the scant literature on aging-related changes in pre-attentive sensory-cognitive processing in the somatosensory modality.

## AUTHOR CONTRIBUTIONS

Juho M. Strömmer, Ina M. Tarkka and Piia Astikainen designed the experiment. Juho M. Strömmer recorded and analyzed the data. The interpretation of data was done by Piia Astikainen, Ina M. Tarkka and Juho M. Strömmer. The manuscript was prepared by Juho M. Strömmer and revised by Piia Astikainen and Ina M. Tarkka. All of the authors approve the final version of the manuscript to be published.

## Conflict of Interest Statement

The authors declare that the research was conducted in the absence of any commercial or financial relationships that could be construed as a potential conflict of interest.
